# Mental and Physical Symptoms of Female Rural Workers: Relation between Household and Rural Work

**DOI:** 10.3390/ijerph120911037

**Published:** 2015-09-07

**Authors:** Marta Regina Cezar-Vaz, Clarice Alves Bonow, Mara Regina Santos da Silva

**Affiliations:** 1 School of Nursing, Federal University of Rio Grande, Rio Grande, RS 96203-900, Brazil; 2 Graduate Program on Nursing, Federal University of Pampa, Uruguaiana, RS 97500-970, Brazil; E-Mail: claricebonow@unipampa.edu.br; 3 School of Nursing, Federal University of Rio Grande, Rio Grande, RS 96203-900, Brazil; E-mail: marare@brturbo.br

**Keywords:** mental health, workload, occupational environment, home environment, farm workers, female work

## Abstract

This study aimed to investigate the relations among mental disorders, physical discomfort, household work and farm work among women. We conducted a cross-sectional study based on the administration of a structured questionnaire to 182 female farm workers. The data were analyzed by means of Poisson regression, where the significance level was set to 5%. Results indicated that 111 (61%) participants reported work-related mental disorders and physical discomfort was reported by 160 (87.9%). The participants with mental disorders and at least moderate levels of physical discomfort reported significantly higher levels of physical demand, time working (temporal demand), total effort and frustration with regard to farm work, in addition to significantly higher levels of frustration with regard to housework. Women with moderate or greater levels of both physical discomfort and frustration with farm work increased the chances of mental disorders. The results illustrate the complex challenge for health professionals in caring for women with mental disorders and physical discomforts related to their farm work, in the context of both the farm and domestic work demands these workers experience.

## 1. Introduction

Mental disorders are defined as a series of symptoms or behaviors associated in most cases with distress and with interference with personal functions that conform to the International Statistical Classification of Diseases and Related Health Problems, 10th Revision (ICD-10) [1]. These conditions include diseases such as depression, bipolar affective disorder, schizophrenia, anxiety disorders, stress, disorders related to substance abuse, disorders of the sleep-wake cycle, mental deficiencies and developmental and behavioral disorders [2]. Such disorders might be related to work as a function of the type of work activities and the conditions under which work is performed [3]. In this regard, mental disorders are described as being more frequent in rural settings than urban environments [4]. More specifically, studies conducted in Taiwan [5], India [6], Australia [7] and Ethiopia [8] found a high prevalence of mental disorders among female rural (primarily agricultural) workers. Most of these studies focus on the relationships of women with their fetuses and newborns (pre- and post-natal depression) [5,7,8] as well as with their partners (domestic violence) [6]. Although fewer in number, some studies described high prevalence rates of mental disorders among women in association with rural work [9,10,11].

A study conducted in the United States found a higher prevalence of depressive symptoms among agricultural workers compared to other women from the same rural area who had different jobs (teachers, caregivers and maids, among others) [9], which indicates that mental disorders might be more related to the type of occupation than the area of residence. A study conducted in Australia described the prevalence of psychological distress, depression and anxiety in three rural communities to identify risk levels according to gender and age. The authors found that the highest rates of psychological distress, depression and anxiety occurred among people between the ages of 45 and 54, without any differences between genders [10]. A study performed with the population of a rural district in Kenya (men and women) found that the prevalence of mental disorders was 10.8%, also without any differences between genders. The observed mental disorders included depression, anxiety and panic disorder [11]. This evidence shows that rural populations, especially female agricultural workers, exhibit work-related mental disorders.

Agricultural work is characterized by intensive and repetitive physical effort [12] that might cause physical discomfort. Physical discomfort is associated with the physical load imposed by the activity, thereby implying that physical discomfort is directly related to the musculoskeletal load [13]. Several studies showed that the physical load imposed by agricultural work is able to cause physical discomfort. A study investigating work-related musculoskeletal disorders conducted with male and female agricultural workers found that workers’ main complaint was low-back pain, with weeding being characterized as the most strenuous activity for females [12]. In another study that assessed safety and health issues among male and female agricultural workers, the participants reported suffering from physical discomfort as a function of the activities they performed: both the heavy ones, such as weeding and carrying heavy items (water buckets, for instance), which caused low-back pain; and the lighter ones, such as creating bouquets of flowers, which caused pain in the hands, wrists and fingers [14].

Housework is another factor that contributes to increasing physical discomfort among female farm workers. A study on the distribution of housework and health among married female workers with small children showed that increasingly unequal distribution of household duties was associated with higher levels of work-related stress, greater work-related fatigue, lower levels of general well-being, and a lower satisfaction with their general life situation. In addition, women with an unequal distribution of household work had insufficient time to sleep, relax and work out [15].

Therefore, although the literature on mental disorders did not differ by gender, studies on physical discomforts show that farm work can be more stressful for women. Additionally, unequal distribution of housework among might lead to increased stress, decreased sleep, and increased dissatisfaction among female workers. We conducted this study to investigate the relationship of both housework and agricultural work (individually and jointly) on the physical and mental health of female farm workers.

## 2. Methods

The present cross-sectional study on the relationship among mental disorders, physical discomfort, housework and agricultural work was performed in the interior of the state of Rio Grande do Sul, Brazil. The sample size was calculated using the StatCalc tool of Epi Info^®^ (version 6.04, CDC, Atlanta, GA, USA). The overall population was considered, without knowledge of the number of female farm workers, with a 5% margin of error, 95% confidence level and 5% losses. The selection of female farm workers occurred in non-probabilistic, convenience sampling, in the area covered by the study. The inclusion criteria were residing in the area where the study was conducted, being 18 years of age or older, and working with agricultural produce. The single exclusion criterion was not performing farm work.

The sample comprised 182 female farm workers, who were recruited one-on-one personally by investigators in women’s residences; from the total potential population, 35 women could not be located after a minimum of five attempts, and a further 13 explicitly refused to participate. We emphasize that was used official vehicle of the Federal University of Rio Grande to conduct the women recruitment as a means of ensuring interviewers’ security and streamlining the recruitment process.

The tasks performed by the participants covered the full process of horticultural work: preparing the soil; growing plants (lettuce, tomato, arugula, kale, chayote and cucumber); preparing seedlings; and caring for, watering, storing, and harvesting crops. The participants were also responsible for the housework at their homes, which included the preparation of meals (planning, organizing and cooking) and performance of household chores (organization, cleaning and laundry).

Data collection was performed by means of interviews following a structured questionnaire, including questions on demographics (age, skin color, marital status, status of literacy and educational level); their housework and farm work conditions (household chores, length of agricultural work experience, number of hours worked daily in the fields, type of agricultural property, property size, monthly income, workload at home and in the fields); and the presence/absence of work-related mental disorders and physical discomfort.

Mental disorders were assessed by self-reported medical diagnosis meeting the definitions of the Health Ministry [16] and ICD-10 [1] generalized anxiety disorder (F41.1)—anxiety that is generalized and persistent but not restricted to, or even strongly predominating in, any particular environmental circumstances (*i.e.,* it is “free-floating”); dominant symptoms are variable but include complaints of persistent nervousness, trembling, muscular tensions, sweating, lightheadedness, palpitations, dizziness and epigastric discomfort. Depressive episodes (F32)—the patient suffers from a low mood and exhibits less energy, activity, interest and concentration; sleep is usually disturbed and appetite is diminished; ideas of guilt or worthlessness are often present. Acute stress reaction (F43.0)—a transient disorder that develops in an individual without any other apparent mental disorders in response to exceptional physical and mental stress and that usually subsides within hours or days; symptoms include an initial “dazed” state with some constriction of the field of consciousness and narrowing of attention, inability to comprehend stimuli and disorientation. Nonorganic disorder of the sleep-wake schedule (F51.2)—characterized by a lack of synchrony or an inappropriate sleep-wake cycle, resulting in complaints of either insomnia or hypersomnia. Panic disorder (episodic paroxysmal anxiety disorder) (F41.0)—characterized by recurrent severe anxiety (panic) attacks that are unpredictable; dominant symptoms include palpitations, chest pains, choking sensation, dizziness and feelings of unreality.

Physical discomfort was assessed based on the participants’ self-reported symptoms (pain, cramps and hypoesthesia), which are in line with the symptoms for work-related musculoskeletal and connective tissue diseases described by the Health Ministry [16]. Pain appears spontaneously or in conjunction with passive or active motions; cramps are defined as spasmodic muscle contractions; and hypoesthesia consists of formication with decreased strength.

The National Aeronautics and Space Administration Task Load Index (NASA-TLX) [17] was used to measure the agricultural and household workload. This workload assessment tool were described in studies conducted by Hoonakker *et al.* [18] and Noyes and Bruneau [19]. The household and agricultural workloads were assessed according to the following types of demands: mental (thinking, choosing, calculating and making decisions), physical (pulling, pushing, carrying and weeding), temporal (the amount of time needed to perform an activity), performance (the quality and agility with which activities are performed), total exertion (physical and mental requirements to perform activities) and level of frustration (motivation, satisfaction, discouragement and irritation) [20]. Participants select a number from 1 to 20 to determine the workload of each demand; higher scores indicate a higher workload. The reliability of these variables was assessed using Cronbach’s alpha, which was 0.97 for the household workload and 0.67 for the agricultural workload.

**Figure 1:**
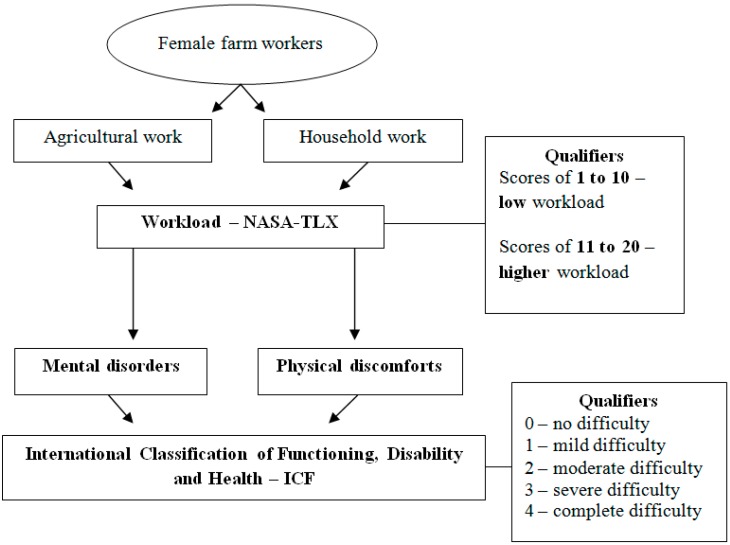
Characterization of agricultural and household demands, workload, mental disorders, and physical discomforts.

The work-related mental disorders and physical discomfort were further qualified using standardized questions based on the International Classification of Functioning, Disability and Health (ICF) [21]. The ICF was approved in 2001 by the by the World Health Assembly and describes the functional status associated with the health of groups and / or individuals [22]. For this, ICF using qualifiers which indicate the extent of mental disorder or physical discomfort restriction represent. The qualifiers employing the following values: 0 (no difficulty), 1 (mild difficulty: 5%–24%), 2 (moderate difficulty: 25%–49%), 3 (severe difficulty: 50%–95%) and 4 (complete difficulty: 96%–100%). For better understanding a scheme were included (Figure 1).

Statistical analysis was performed with the Statistical Package for Social Sciences (SPSS 20.0, IBM, Armonk, NY, USA). Descriptive analysis was performed (mean, standard deviation, median and interquartile range). The Mann-Whitney test was used to analyze differences in the participants’ basic characteristics, physical discomfort and workload (household and agricultural) among female workers with and without mental disorders. Student’s t-test was used to compare means between the groups for independent samples, Pearson’s chi-square test and Fisher’s exact test were used to compare proportions, and Poisson regression was performed to control for confounding factors. Variables that exhibited *p*-values < 0.20 in the bivariate analysis were included in the regression model. The significance level was set to 5% (*p* ≤ 0.05).

All subjects gave their informed consent for inclusion before they participated in the study. The study was conducted in accordance with the Declaration of Helsinki, and the protocol was approved by the Ethics Committee of Federal University of Rio Grande (026/2013).

## 3. Results

The average age of the sample was 48.9 years. With regard to ethnicity, 167 participants (91.8%) were white, eight (4.4%) were brown-skinned, six (3.3%) were black, and one (0.5%) was of Indian ethnicity. In terms of marital status, 160 (87.9%) women were married, 12 (6.6%) were single, eight (4.4%) were widowed, and two (1.1%) were divorced. A total of 120 participants (65.9%) had not completed elementary school, 15 (8.2%) had completed elementary school, 11 (6.0%) had not completed secondary school, 17 (6.0%) had completed secondary school, five (2.7%) had not completed higher education, one (0.5%) had completed higher education, one (0.5%) had a graduate degree, and 12 (6.6%) were illiterate.

Most of the women (89; 49.2%) worked at their own properties, which belonged to their families in 61 (33.7%) cases, were ceded properties in 20 (11%) cases, were leasehold estates in nine (5%) cases, and were financed in two (1.1%) cases. The median property size was seven hectares. The average monthly family income of the participants was BRL 1,500.00, and the *per capita* income was BRL 500.00. The mean agricultural work experience was 32 years, with an average of 6.7 h of work per day.

Most participants (176; 96.7%) performed agricultural and household work; 42 female farm workers (23.07% of all female farm workers) had her husband’s help in housework. Work-related mental disorders were reported by 111 female farm workers (61%). The most prevalent mental problem was generalized anxiety disorder (69; 37.9%), followed by acute stress reaction (67; 36.8%), nonorganic disorder of the sleep-wake schedule (55; 30.2%), depressive episodes (33; 18.1%) and panic disorder (episodic paroxysmal anxiety disorder; 9; 4.9%). The mental disorders were described as mild in 32 cases (28.8%), moderate in 48 cases (43.2%), severe in 20 cases (18%) and complete in 11 cases (9.9%).

Most of the participants (160; 87.9%) reported experiencing physical discomfort. The experienced physical pain was distributed as follows: upper body (145; 79.7%), lower limbs (87; 47.8%) and upper limbs (54; 29.7%). A total of 46 (25.3%) participants reported experiencing cramps in their lower limbs, whereas five (2.7%) reported cramping in the upper limbs and upper body. A total of 48 (26.4%) women reported experiencing numbness in the upper limbs, 16 (8.8%) experienced numbness in the lower limbs, and two (1.1%) experienced it in the upper body. With regard to the classification of physical discomfort, 20 (12.5%) participants described it as mild, 70 (43.8%) described it as moderate, 42 (26.3%) described it as severe, and 25 (15.6%) described it as complete; three (1.9%) participants did not know how to qualify their physical discomfort.

The analysis of the relationship between the presence or absence of mental disorders was adjusted in accordance with the participants’ demographic data, working conditions and physical discomfort; a significant relationship was found between the presence of mental disorders and skin color (*p* = 0.044) and at least a moderate level of physical discomfort (*p* < 0.001). White women with at least moderate physical discomfort exhibited a higher probability of having a mental disorder. All participants described similar working conditions. Because the number of participants who reported not experiencing any physical discomfort was too small, it was categorized as follows: (1) no/mild discomfort and (2) moderate/severe/complete discomfort. After dichotomizing the outcome, its relation with the other study variables was examined. Physical discomfort was found to be significantly related to age range (*p* = 0.026), skin color (*p* = 0.050) and property type (*p* = 0.007). White women between the ages of 40 to 59 who worked at properties that did not belong to them or their families exhibited higher probabilities of experiencing physical discomfort. Given that most women performed both agricultural and household work, the relationship of the scores attributed to both the agricultural and household workloads with the presence or absence of mental disorders and physical discomfort was investigated (Table 1).

**Table 1:** Relationship of presence or absence of mental disorders and physical discomfort with household and agricultural workloads among female farm workers from the interior of the state of Rio Grande do Sul, Brazil.

Agricultural and Household Workload	Presence of Mental Disorder	Absence of Mental Disorder	P Value	Presence of At Least Moderate Physical Discomfort	No/Mild Physical Discomfort	P Value
	*n* = 111 (%)	*n* = 71 (%)		*n* = 137 (%)	*n* = 42 (%)	
	Mean ± SD	Mean ± SD		Mean ± SD	Mean ± SD	
**Agricultural workload**						
Mental demands	12.7 ± 6.0	11.2 ± 6.0	0.100	12.7 ± 6.0	10.0 ± 5.7	0.009
Physical demands	16.7 ± 4.4	14.3 ± 5.1	0.001	16.3 ± 4.6	14.1 ± 5.1	0.008
Temporal demands	14.5 ± 5.3	11.8 ± 5.6	0.001	14.1 ± 5.5	11.3 ± 5.1	0.004
Performance	14.3 ± 5.9	15.4 ± 4.8	0.183	14.6 ± 5.8	15.2 ± 4.9	0.513
Total exertion level	15.9 ± 4.7	14.4 ± 5.2	0.045	16.1 ± 4.4	12.6 ± 5.7	0.001
Frustration level	13.2 ± 6.9	9.6 ± 7.5	0.001	12.7 ± 7.2	9.2 ± 7.2	0.008
**Household workload**						
Mental demands	12.7 ± 5.5	12.2 ± 5.9	0.596	13.2 ± 5.3	10.9 ± 6.2	0.025
Physical demands	13.6 ± 5.7	11.7 ± 6.1	0.032	13.4 ± 5.9	11.5 ± 5.9	0.087
Time demands	12.4 ± 6.0	11.9 ± 6.5	0.611	12.8 ± 6.3	10.6 ± 5.8	0.054
Performance	14.6 ± 5.4	16.2 ± 4.7	0.043	14.8 ± 5.2	16.3 ± 5.2	0.130
Total exertion level	15.4 ± 4.8	14.4 ± 5.5	0.189	15.7 ± 4.6	13.0 ± 6.0	0.010
Frustration level	11.9 ± 7.2	8.1 ± 6.6	0.001	11.3 ± 7.3	8.0 ± 6.6	0.012

With regard to the agricultural workload, the participants with mental disorders reported significantly higher levels of physical demand, time working (temporal demand) and total exertion, and frustration. With regard to household workload, this group also reported significantly higher physical exertion, performance and frustration levels. Participants with at least moderate levels of physical discomfort reported higher levels of mental, physical, temporal and total exertion; and frustration in relation to agricultural work in addition to higher levels of mental and total exertion as well as frustration with regard to housework. Following inclusion in the multivariate Poisson regression model, the participants with higher levels of frustration with agricultural work had a 2% higher chance of exhibiting mental disorders, whereas the prevalence of mental disorders among the women experiencing at least moderate levels of physical discomfort was 106% higher than that among the workers with no/mild physical discomfort. In addition, at least moderate physical discomfort remained significantly associated with the variables of property type, per capita income and total exertion demands imposed by agricultural work. The prevalence of at least moderate physical discomfort was 26% higher among the women who worked in properties that did not belong to them or their families. In contrast, higher income reduced the probability of experiencing physical discomfort by 2%, whereas an increase of one level in the degree of total exertion associated with household work increased the prevalence of at least moderate physical discomfort by 4% (Table 2).

**Table 2:** Poisson regression analysis to assess factors associated with mental disorders and physical discomfort among female farm workers from the interior of the state of Rio Grande do Sul, Brazil.

Variables	PR (95% CI)	Value
**Mental Disorders**		
Frustration with farm work	1.02 (1.00-1.04)	0.035
At least moderate level of physical discomfort	2.06 (1.27-3.35)	0.004
**Physical Discomfort**		
Type of property	1.26 (1.12-1.41)	< 0.001
Per capita income	0.98 (0.95-0.99)	0.032
Total exertion imposed by farm work	1.04 (1.02-1.06)	< 0.001

PR: prevalence ratio; 95% CI: 95% confidence interval.

## 4. Discussion

The type of mental disorder most frequently reported by the interviewed farm workers corroborates the findings of a study conducted with men and women from an agricultural district in Kenya [11], indicating that a combination of generalized anxiety with depressive episodes is the most common mental problem among farm workers. Similarly, the indication of the moderate qualifier was the same as that found in a study on the occurrence of depressive symptoms among farm workers [9]. We cannot specify why generalized anxiety was the most frequently mental disorder because the exact causes of this disorder is not fully understood [23], however, it is know that prevalence of generalized anxiety disorder in women is about twice that of men, which may explain the higher frequency [24].

Most participants reported experiencing physical discomfort. A study conducted with male and female farm workers to detect musculoskeletal problems and physical discomfort found that the upper body was the most affected body part [12], similar to the results of the present study, in which physical pain was most commonly reported in the upper body. In the previously mentioned study on depressive episodes [9], most (46.5%) of the investigated female farm workers described the intensity of their discomfort as mild, whereas 39.5% described it as severe. In the present study, most participants described their physical discomfort as moderate, which is in line with the results of the previously mentioned study [9].

The analysis showed a relation between the presence of mental disorders and at least moderate physical discomfort (*p* < 0.001), whereas the regression analysis indicated that the prevalence of mental disorders among participants with at least moderate physical discomfort was 106% higher relative to workers with no/mild discomfort. These findings support the results of a study that assessed mental disorders among farm workers and showed that workers with physical disorders are the most affected by mental disorders [9]. A study performed in Denmark with individuals with physical discomfort sought to establish whether anxiety and fear were relevant to the seeking of care for back pain; the results showed that anxiety was associated with back pain among women [25].

When physical discomfort was included in the regression model, it proved to be associated with the variables of property type, per capita income and total exertion related to farm work. The prevalence of at least moderate physical discomfort exhibited by the participants who worked in properties that did not belong to them or their families was 26% higher. We hypothesize that the participants who were not property owners, as well as those who worked in family properties, had to exert excessive effort to earn a living and thus exhibited physical discomfort. These findings corroborate the results of a study conducted with a Korean population [26], which found that the prevalence of musculoskeletal, and therefore physical, discomfort was higher among women with lower incomes, similar to the women in the present study, who have an average family income of BRL 1500.00 and a *per capita* income of BRL 500.00. In addition, higher income levels reduced the chance of experiencing physical discomfort by 2%, that is, income served as a protective factor against physical discomfort; this is in line with the results of a study on individuals from agricultural areas in China, which sought to identify the variables that explained the use of health services [27].

Greater family income is a characteristic associated with increased use of health services, whereby income serves as a protective factor of human health because people with greater incomes seek healthcare more often. In the present study, the level of total exertion demanded by farm work increased the prevalence of at least moderate physical discomfort by 4%. It should be noted that total exertion considers the physical and mental requirements [20] for the female farm workers to perform field work, that the required physical and mental exertion to perform farm work is associated with physical discomfort.

Female workers with mental disorders reported significantly higher levels of physical, temporal and total effort as well as frustration with regard to farm work. Farm work is known to entail a heavy workload. A report on the psychosocial safety of Australian workers indicated that the physical demands of work are higher among rural than urban workers, which contributes to the greater emotional exhaustion of the former [4] as well as to the increase of total work-related exertion. With regard to the time demands of field work, in a study that investigated horticultural workers’ safety and health, the participants noted that after a long day of working in the fields, they still had to prepare the vegetables that would be sold the following day and wake up very early the next morning to set up the sales stands at the market. Therefore, it is understood that time demands are highly significant because rural work is continuous and intensive [14]. With regard to frustration, a study performed in Peru that assessed gender roles and identities in rural development found that a lack of money is a factor that increases the levels of frustration and anxiety among rural workers [28]; hence the workload relative to frustration is greater for those female workers.

With regard to housework, the participants reported significant levels of physical and performance demands as well as frustration. Women are typically more involved with housework than men [29]. Housework comprises activities related to cleaning and organizing the home. A study performed with agricultural workers showed that women had a higher number of total working hours than men due to the combination of agricultural and household work [30]. The longer working hours of female farm workers led to a study with male and female farm workers in the North of India. As a function of social norms, some women do not assert their identities as farm workers but rather seek recognition by focusing their actions on housework [31].

The regression analysis showed that participants with higher levels of frustration towards farm work had a 2% higher probability of exhibiting mental disorders. A study on the global assessment of the environmental and socioeconomic impacts of drought on agricultural workers showed that drought was associated with increased levels of frustration and anxiety – *i.e.*, the mental disorder most often mentioned by the agricultural workers in the present study [32]. It is known that drought has economic and work impacts resulting from decreased profits, production losses and the consequent need to repeat work that had already been performed and was lost. With regard to the participants in the present study, it is not possible to assert that their frustrations toward agricultural work were caused by drought, but regardless of the reasons for their frustration, the impacts of drought are quickly felt and impose a greater workload, which increases physical discomfort and mental disorders in this population. It is known that the set of results remains a complex challenge for health professionals, as regards the possible associations between mental disorders and physical discomforts and the overhead of agricultural and household work which these workers are exposed.

## 5. Conclusions

The most common mental disorder reported by the participants was generalized anxiety, and the most common cause of physical discomfort was pain in the upper body. The mental disorders exhibited by the participants were associated with their frustration with agricultural work as well as at least moderate levels of physical discomfort. Physical discomfort was associated with property type, household and per capita income and the level of total exertion demanded by agricultural work.

Mental disorders should be considered relevant for the development of physical discomfort. In contrast, income was protective against physical discomfort. It is understood that although the present study had some limitations as a function of its cross-sectional design, which did not allow for the investigation of causes (whereas the associations among mental disorders, physical discomfort, household and agricultural work were investigated), it represents a relevant source of information for studies with other designs, as well as for the planning of prevention, promotion and rehabilitation initiatives within the scope of healthcare for rural populations.
